# Effects of Lyophilized Açaí (*Euterpe oleracea*) Supplementation on Oxidative Damage and Intestinal Histology in Juvenile Shrimp *Penaeus vannamei* Reared in Biofloc Systems

**DOI:** 10.3390/ani13203282

**Published:** 2023-10-20

**Authors:** Grecica Mariana Colombo, Robson Matheus Marreiro Gomes, Sonia Astrid Muñoz Buitrago, Juan Rafael Buitrago Ramírez, Alan Carvalho de Sousa Araujo, Fernando Pablo Silva Oliveira, Virgínia Fonseca Pedrosa, Luís Alberto Romano, Marcelo Tesser, Wilson Wasielesky, José María Monserrat

**Affiliations:** 1 Laboratório de Bioquímica Funcional de Organismos Aquáticos (BIFOA), Instituto de Oceanografia (IO), Universidade Federal do Rio Grande—FURG, Rua do Hotel, n° 02, Rio Grande CEP 96210-030, RS, Brazil; roobinho_matheus@hotmail.com (R.M.M.G.); sonymuzb@gmail.com (S.A.M.B.); juanrafaelb25@gmail.com (J.R.B.R.); alandesousa02@hotmail.com (A.C.d.S.A.); 2 Programa de Pós-Graduação em Aquicultura, Instituto de Oceanografía (IO), Universidade Federal do Rio Grande—FURG, Rio Grande CEP 96200-970, RS, Brazil; pablo2fp@gmail.com (F.P.S.O.); vikavet@yahoo.com.br (V.F.P.); luisalbertoromano0@gmail.com (L.A.R.); mbtesser@gmail.com (M.T.); manow@mikrus.com.br (W.W.); 3 Laboratório de Imunologia e Patología de Organismos Aquáticos, Instituto de Oceanografía (IO), Universidade Federal do Rio Grande—FURG, Rio Grande 96200-970, RS, Brazil; 4 Laboratório de Nutrição de Organismos Aquáticos (LANOA), Instituto de Oceanografía (IO), Universidade Federal do Rio Grande—FURG, Rio Grande 96200-970, RS, Brazil; 5 Laboratório de Carcinocultura, Instituto de Oceanografia (IO), Universidade Federal do Rio Grande—FURG, Rio Grande 96200-970, RS, Brazil; 6 Instituto de Ciências Biológicas (ICB), Universidade Federal do Rio Grande—FURG, Av. Itália km 8 s/n, Cx. P. 474, Rio Grande CEP 96200-970, RS, Brazil

**Keywords:** antioxidant, bioactive compounds, polyphenols

## Abstract

**Simple Summary:**

Açaí (*Euterpe oleracea*) is a fruit of Amazonian origin rich in phenolic compounds, which are responsible for the high antioxidant capacity of this fruit. In addition to contributing to the maintenance of water quality, bioflocs serve as a supplementary food for farmed shrimp. Because they also contain bioactive compounds with antioxidant properties, the consumption of bioflocs by shrimp helps maintain the balance of physiological functions and the antioxidant status of these crustaceans. Studies have demonstrated the ability of bioflocs to incorporate exogenous antioxidants into a biofloc technology system (BFT). In this context, the antioxidants present in açaí, when added to the cultivation of juvenile shrimp *Penaeus vannamei* in a BFT system, can be assimilated by bioflocs, serving as a vector for the transfer of these bioactive compounds to the shrimp.

**Abstract:**

The objective of this was to evaluate the ability of bioflocs to assimilate and transfer antioxidant compounds present in açaí *Euterpe oleracea* to juvenile *Penaeus vannamei* shrimp grown in a biofloc system. Juvenile shrimp were distributed into four treatment groups (control, 5, 20, and 80 mg açaí L^−1^), containing 31 shrimps/tank (90 L), and cultivated for 30 days. Every 24 h throughout the experimental period, the respective açaí concentrations were added directly to the cultivation water. The bioflocs and hepatopancreas lost their antioxidant capacity with increasing concentrations of açaí; however, lipid damage was mitigated after treatment with 20 mg of açaí L^−1^ (*p* < 0.05). The application of 20 mg açaí L^−1^ increased the mean height and area of the middle intestinal microvilli (*p* < 0.05). Mortality and protein and lipid damage in shrimp muscle increased with daily administration of 80 mg açaí L^−1^ (*p* < 0.05). It is concluded that the bioflocs were able to assimilate the antioxidants present in açaí and transfer them to the shrimp, and the administration of 20 mg açaí L^−1^ presented the best performance, demonstrating the possibility of its application in the cultivation of *P. vannamei* in a biofloc system.

## 1. Introduction

Açaí (*Euterpe oleracea*) is a palm tree endemic to the Amazon region, in the states of Pará and Amazonas, where the world’s largest production of açaí is concentrated, with a culture corresponding to 87.5% of the Brazilian national market [1,2]. Among the various processed açaí genres, the most consumed part is the fruit, which is the main raw material for the production of juices and pulps [3,4]. Owing to its nutritional composition, açaí has gained notoriety in the financial and consumer markets, becoming the object of different studies because of its high content of proteins, lipids, fibers, and bioactive compounds with high antioxidant capacity [5,6,7].

The antioxidant activity observed in the fruit is directly related to the phenolic compounds, mainly from the flavonoid group. Among these, anthocyanins, phenolic acids, flavonols, and carotenoids are the most common, with anthocyanins being the main pigment responsible for the purple color of açaí and its significant antioxidant capacity [6]. These compounds are associated with nutritional, anti-inflammatory, antimicrobial, anticancer, cardiovascular, neurological, and antioxidant effects in humans and animals [6,8,9,10].

The production of aquatic organisms is subject to variations in different environmental factors common to cultivation, which can generate stress conditions in animals and contribute to the production of reactive oxygen species (ROS) and the reduction in antioxidant activity [11,12,13,14]. Although animals possess endogenous antioxidant defenses to cope with oxidative stress, a considerable amount of metabolic energy is required to improve or prevent this imbalance [15]. In this context, the supplementation or inclusion of food additives with a high antioxidant capacity in the diet of fish and shrimp serves as a strategy to increase the resistance of these organisms to stressful conditions [13,14,16,17].

Although scarce, studies on the application of açaí in shrimp diets have shown promising results regarding chemoprevention. The inclusion of 10% (*w*/*w*) açaí in the diet of *P. vannamei* shrimp cultivated in clear water showed a chemoprotective effect against ammonia exposure, resulting in an improvement in the antioxidant defense system and the attenuation of histopathological damage in the hepatopancreas [10], in addition to conferring protection against lipid damage in this same organ in shrimps challenged with saxitoxin [18].

In contrast to conventional production systems, the biofloc technology system (BFT) is characterized by minimal or no water exchange, reduced effluent release, high productivity, and biosafety [19,20]. Bioflocs have 18–43% protein in their composition and are responsible for contributing up to 29% of shrimp feed, contributing to reducing artificial feeding rates and improving feed conversion rates [21,22]. Because they contain proteins, fatty acids, minerals, vitamins, digestive enzymes, and antioxidant molecules, bioflocs not only serve as supplementary food for the growth of farmed shrimp but are also natural and abundant sources of bioactive compounds that contribute to the physiological health of crustaceans [22,23,24,25].

Recently, studies have been conducted on the application of lyophilized açaí in biofloc systems. In a study by Silva et al. [26], açaí was included directly in the diet composition of *P. vannamei* juveniles, at four concentrations tested (control, 2.5%, 5.0%, and 10.0% *w*/*w*). Although there were no effects on growth, the accumulation of flavonoids in the bioflocs and gills of the 5.0% açaí treatment group demonstrated the ability of bioflocs to absorb the antioxidants present in this fruit. Balancing and elaborating a diet that includes supplements require considerable effort. Therefore, the direct addition of açaí to cultivation appears to be more practical [27]. Studies by Leon et al. [25] and Colombo et al. [27] were pioneers in enriching bioflocs with antioxidants. Colombo et al. [27] reported the use of Amazonian fruit açaí as a natural source of antioxidants. The authors tested the daily addition of four concentrations of lyophilized açaí (control, 5, 20, and 80 mg of açaí L^−1^) to the culture of *P. vannamei* post-larvae. In addition to water quality parameters, such as maintaining average water alkalinity levels above 100 mg CaCO_3_ L^−1^, açaí improved the antioxidant status of bioflocs, mainly seen in the significant increase in the survival rate of the post-larvae of all treatment groups that received this fruit, which is critical in the production of the post-larvae in the nursery phase.

Therefore, due to the high antioxidant content and protection conferred by açaí to crustaceans, based on the same principle as that provided by Colombo et al. [27] for post-larvae, the present study aimed to continue the application of lyophilized açaí in a biofloc system for rearing juveniles of *P. vannamei*. Through biochemical and histological analyses and zootechnical parameters, we sought to evaluate the ability of shrimp to absorb antioxidants present in açaí, as well as the role of bioflocs in the assimilation and transfer of such bioactive compounds via food.

## 2. Materials and Methods

### 2.1. Shrimp Maintenance and Experimental Design

*Penaeus vannamei* shrimps with a medium initial weight of 0.96 to 1.10 g were obtained from a shrimp farming system at the Marine Aquaculture Station (EMA) of the Federal University of Rio Grande—FURG, located at Cassino Beach, Rio Grande, RS, Brazil. Lyophilized açaí (*Euterpe oleracea*) used in the present study were obtained from Amazônia Comércio de Açaí Liofilizado e Exportação LTDA, Belém, PA, Brazil.

The biofloc inoculum used in the experiment was collected from a super-intensive cultivation system of Pacific White Shrimp *P. vannamei* and distributed in four tanks (90 L of usable volume) without the presence of shrimp (pH 7.94, total ammonia 0.21 mg/L, nitrite 0.07 mg/L, nitrate 80.00 mg/L, phosphorus 4.30 mg/L, alkalinity 137.50 mg/L, SST 310 mg/L, and salinity of 30 ppt). For seven days, the bioflocs were enriched with three concentrations of açaí (5, 20, and 80 mg of açaí L^−1^), beyond the control treatment, added directly to the water in each tank every 24 h. These açaí concentrations were previously tested by Colombo et al. [27] for the cultivation of *P. vannamei* post-larvae in a biofloc system.

After biofloc enrichment, juvenile shrimps were stored in 12 circular polyethylene tanks with a useful volume of 90 L each, disposed in four treatments (control, 5, 20, and 80 mg of açaí L^−1^) in triplicate, containing 31 shrimps per tank. In each experimental unit, 67.5 L of seawater treated with a chlorine solution (10 ppm) and dechlorinated with ascorbic acid (1:1 *w*/*w*) and 22.5 L of biofloc inoculum previously enriched with açaí were used. The inoculum volume in each tank corresponded to 25% of the useful volume of the experimental units according to the methodology of Krummenauer et al. [20]. The experiment lasted 30 days and every 24 h, during the experimental period, the respective concentrations of lyophilized açaí were added directly to the cultivation water. Shrimps were fed twice a day with commercial feed GUABI^®^ 35% crude protein (moisture 100 g, ethereal extract 100 g, crude fiber 40 g, and minerals 140 g), according to the protocol described by Jory [28]. Weekly biometrics (*n* = 12) were performed to adjust the ration provided. The aeration used during the enrichment of the inoculum with açaí and in the cultivation of shrimp was provided through aerotubes. The study was carried out in the laboratory with a photoperiod fixed at 12 h L/12 h D and a temperature and mean salinity of 29.80 °C ± 0.56 and 26.68 ppt ± 1.46, respectively.

**Figure 1:**
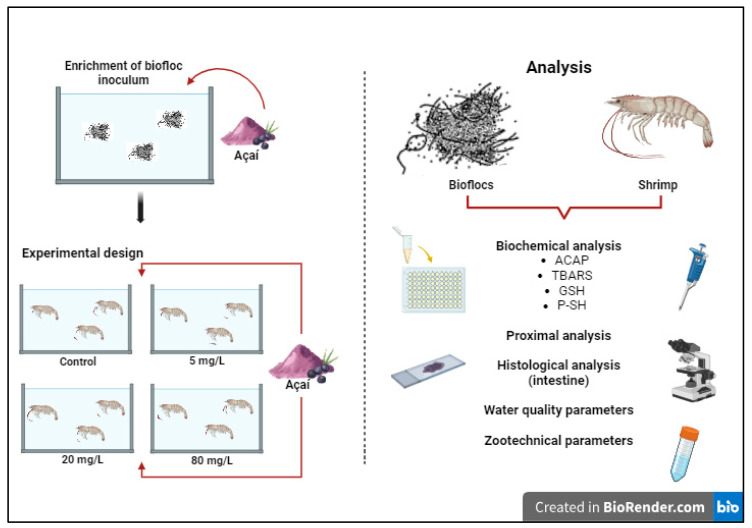
Experimental groups and analyses used in this study. ACAP: total antioxidant capacity against peroxyl radicals. TBARS: thiobarbituric acid reactive substances. GSH: reduced glutathione. P-SH: Protein-associated sulfhydryl groups. Created with BioRender.com.

Daily dissolved oxygen and temperature were measured using a multiparameter digital oximeter (YSI^®^-550A), and pH was measured using a digital pH meter (Alfakit, AT 315 SP). Total ammonia (TAN) (NH_3_ + NH_4_^+^), nitrite (N-NO_2_^−^), and nitrate (N-NO_3_^−^) were monitored following the protocols of UNESCO [29], and alkalinity [30], salinity (Alfakit refractometer), and total suspended solids (TSS) [31] were measured weekly during the study. For ammonia levels above 1 mg L^−1^, cane molasses was added to the experimental units as a carbon source in a proportion of 6 g of carbon for each 1 g of total ammoniacal nitrogen in the water, with the objective of adjusting the C:N ratio of the system [19,32]. When water alkalinity values were below 100 mg CaCO_3_ L^−1^, corrections with sodium bicarbonate (NaHCO_3_) were performed to increase the alkalinity to 200 mg CaCO_3_ L^−1^ [33]. Treatments in which TSS levels exceeded 500 mg/L were used to remove excess total suspended solids, according to the methodology described by Gaona et al. [31]. A schematic drawing demonstrating the experimental groups and analyses performed in this study is shown in Figure 1.

### 2.2. Zootechnical Performance and Proximal Analysis of Shrimp Bioflocs and Muscle

At the start and end of the experiment, individual shrimp were weighed to estimate the following zootechnical parameters:

1. Weight gain (g) = final weight − initial weight.
2. Specific growth ratio = [100% × (Ln final weight − Ln initial weight)/trial duration].
3. Feed conversion ratio = dry feed intake/weight gain.
4. Protein efficiency ratio = weight gain/dry protein intake.
5. Survival (%) = (final number of shrimps/initial number of shrimps) × 100.

Dry matter analysis was performed by drying the biofloc and muscle samples in an oven at 102 °C for 5 h. For ash analysis, the samples were precalcined and then placed in a muffle furnace at 600 °C for 5 h [34]. The crude protein content was determined after sample digestion and nitrogen distillation according to Kjeldahl’s protocol [35]. The lipid content was evaluated using a cold extraction method [36].

### 2.3. Biochemical Analysis

#### 2.3.1. Homogenization of the Samples

At the end of the experiment, to collect the bioflocs, 1 L of water from each experimental unit was collected and added to the Imhoff cones, which were allowed to settle for 20 min. The supernatant was discarded, and the sedimentable solids were centrifuged at 1500× *g* for 15 min at 10 °C to remove excess water and stored in an ultrafreezer at −80 °C [25]. The homogenate of the bioflocs was obtained by sonicating the samples (QSonica Sonicators; Newtown, CT, USA) for 1 min in 30 ppt artificial salt water (Salt Veromix (São Paulo, Brazil), 95%, and 1:1 *w*/*v*), stirring in an orbital shaker at 80 rpm for 3 h and centrifuged at 10,000× *g* for 5 min at 4 °C [27]. The shrimps were weighed individually, euthanized in liquid nitrogen, and the organs (gills, hepatopancreas, and muscle) were collected and homogenized (1:5 *w*/*v*) in a buffer solution for crustaceans (pH 7.2; Tris-base 20 mM; EDTA 1 mM; 0.05 mM MgCl_2_; 5 mM sucrose; and 1 mM KCl). The samples were centrifuged at 20,000× *g* for 30 min at 4 °C, and the supernatants were stored at −80 °C for biochemical analyses. The total protein content of the biofloc and shrimp samples was determined via the biuret method (550 nm) using a microplate reader (Biotek Synergy HT, Santa Clara, CA, USA) [37].

#### 2.3.2. Total Antioxidant Capacity against Peroxyl Radicals (ACAP)

The analysis of the total antioxidant capacity against peroxyl radicals (ACAP) was performed as described by Amado et al. [37]. Samples of bioflocs and shrimp organs were exposed to peroxyl radicals generated by the thermal decomposition of ABAP (2,2-azobis-2-methylpropionamidine dihydrochloride) (Sigma Aldrich, São Paulo, Brazil) at 37 °C. When reacting with 2′,7′-dichlorofluorescein diacetate (H_2_DCF), peroxyl radicals formed a fluorescent compound (DCF), which was detected using a spectrofluorometer (Biotek Synergy HT; Winooski, VT, USA) (excitation: 485 nm; emission: 535 nm). The ACAP was quantified using the relative area with and without ABAP, where a low relative area indicated a higher antioxidant capacity.

#### 2.3.3. Lipid Peroxidation (TBARS)

Lipid peroxidation was measured using thiobarbituric acid reactive substances (TBARS), as described by Oakes and Van Der Kraak [38]. The homogenates (bioflocs: 50 µL; gills: 50 µL; hepatopancreas: 30 µL; muscle: 100 µL) were added to glass tubes, along with 20 µL of BHT solution (butylated hydroxytoluene, 67 µM), 150 µL of solution of acetic acid 20%, 150 µL of TBA solution 0.8% (thiobarbituric acid), 50 µL of distilled water, and 20 µL of SDS 8.1% (sodium dodecyl sulfate), and were subsequently heated for 30 min at 95 °C. After cooling, 100 µL distilled water and 500 µL n-butanol were added to the final solution and centrifuged at 3000× *g* for 10 min at 15 °C. The supernatant was transferred to a microplate, and readings (excitation: 520 nm; emission: 580 nm) were measured using a microplate spectrofluorometer (Biotek Synergy HT, part number 791000). Tetramethoxypropane (TMP, Acros Organics, Geel, Belgium) was used as a standard, and the results were expressed in TMP equivalents per mg of wet tissue.

#### 2.3.4. Reduced Glutathione (GSH)

To measure the concentration of reduced glutathione (GSH), 240 μL the of sample extract (biofloc, gills, hepatopancreas, and muscle) and 28 μL of trichloroacetic acid (TCA 50% *w*/*v*) were added to Eppendorf tubes. After centrifugation at 20,000× *g* at 4 °C for 10 min, 100 μL of the supernatant was added to a transparent microplate, along with 200 μL Tris-Base 0.4 M at pH 8.9, and 10 μL of DTNB (5.5′-dithiobis (2-nitrobenzoic acid)) (Sigma Aldrich; São Paulo, Brazil), and was incubated in the dark at room temperature for 15 min [39]. Absorbance readings (405 nm) were measured using a spectrofluorometer (Biotek Synergy HT, part number 791000) and the concentration was expressed as μmol of GSH per mg of protein.

#### 2.3.5. Protein-Associated Sulfhydryl Groups (P-SH)

The concentration of sulfhydryl groups associated with the protein (P-SH) also followed the protocol described by Sedlak and Lindsay [39]. The protein precipitate from the biofloc and shrimp organ samples formed after GSH centrifugation was resuspended in 240 μL of artificial saline water and homogenization buffer for crustaceans, respectively, and 100 μL of the extract, 160 μL of Tris-Base 0.2 M) at pH 8.2, and 10 μL of DTNB were added to a transparent microplate. The microplate was incubated in the dark at room temperature for 15 min, and absorbance (405 nm) was measured using a spectrofluorometer (Biotek Synergy HT, part number 791000). The final concentration was expressed as μmol of P-SH per mg of protein.

### 2.4. Histological Analysis

Three shrimps were collected per treatment and anesthetized with ice to collect the intestine, which was fixed in 10% formalin (NBF) for 24 h and later transferred to 70% alcohol. Samples of the anterior and middle intestines were processed in LEICA TP 1020 and included in the Paraplast. Intestines were sectioned into cross-sections at a thickness of 5 µm using a LEICA RM2245 rotary microtome and stained with hematoxylin–eosin (Harris hematoxylin), as suggested by Luna [40] and Romano and Pedrosa [41]. Histological slides were viewed under a Zeiss Primo Star optical photo microscope with an AxioCam ERc 5s camera (Göttingen, Germany). AxioVision LE 4.8.2 SP2 software (Göttingen, Germany) was used to analyze the photographs. The height, width, and area of intestinal microvilli were measured. Histological analysis was performed according to the protocol of Bullerwell et al. [42], in which four photos were taken per intestine (middle and posterior) of the shrimp, totaling 12 photos per treatment of each intestine. In each photograph, three microvilli were randomly selected to remove the established parameters. All measurements were performed using ImageJ Software 1.53.

### 2.5. Statistical Analysis

The homogeneity of the variances of the different treatments was evaluated using the Levene test, and the normality of the data distribution was evaluated using the Shapiro–Wilk test. After verifying the assumptions, all data were subjected to a mixed model analysis of variance (ANOVA), with the concentration of lyophilized açaí added to the cultivation of *L. vannamei* in the BFT system as the fixed factor and the experimental units of each treatment as the random factor [43]. Possible significant differences between treatments were detected using the Newman–Keuls test, and the significance level was set at 0.05.

## 3. Results

The results referring to the water quality parameters are listed in Table 1. During the experimental period, the physicochemical parameters of the water, such as dissolved oxygen, temperature, nitrite, and phosphorus, did not show statistical differences between the treatments (*p* > 0.05). The tanks that received 20 and 80 mg açaí L^−1^ showed an increase in mean pH values compared to the control treatment and 5 mg açaí L^−1^ (*p* < 0.05). The ammonia concentration increased in the 80 mg açaí L^−1^ treatment compared to the other treatments (*p* < 0.05); however, the nitrate concentration decreased significantly in the same treatment (*p* < 0.05). An increase in alkalinity was observed at concentrations above 20 mg açaí L^−1^ compared to the control treatment and 5 mg açaí L^−1^ (*p* < 0.05). The mean values of the total suspended solids increased proportionally with the concentration of lyophilized açaí added to the BFT system (*p* < 0.05).

The results of the proximal analysis of biofloc and shrimp muscles are presented in Table 2. The ash content of the bioflocs decreased proportionally with an increase in açaí concentration (*p* < 0.05). For shrimp muscle, no changes were observed in the content of dry matter, lipids, and ash (*p* > 0.05), but the protein content of the 5 mg açaí L^−1^ treatment was higher than that of the 20 and 80 mg açaí L^−1^ treatments (*p* < 0.05). Regarding the zootechnical parameters, only the survival rates showed a statistically significant difference (Table 3). The daily administration of 80 mg açaí L^−1^ resulted in higher shrimp mortality than the other treatments (*p* < 0.05).

**Figure 2:**
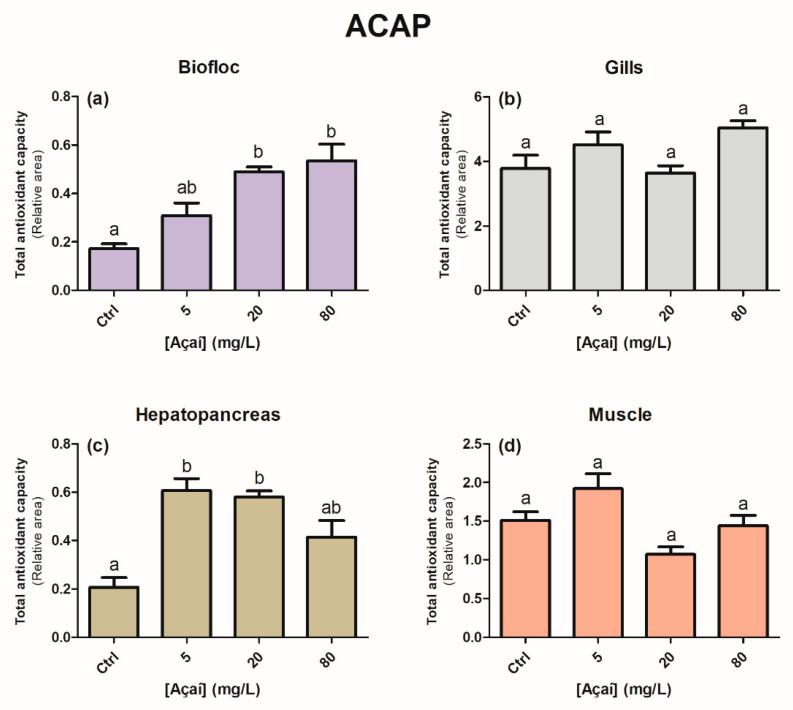
Values of total antioxidant capacity against peroxyl radicals (expressed in relative area) in the bioflocs (**a**), gills (**b**), hepatopancreas (**c**), and muscle (**d**) of *Penaeus vannamei* shrimp after the addition of different concentrations of lyophilized açaí for 30 days. Values are expressed as mean ± 1 standard error (*n* = 12). Different letters indicate statistical differences between the concentrations of lyophilized açaí according to the Newman–Keuls test at a significance level of 0.05.

**Figure 3:**
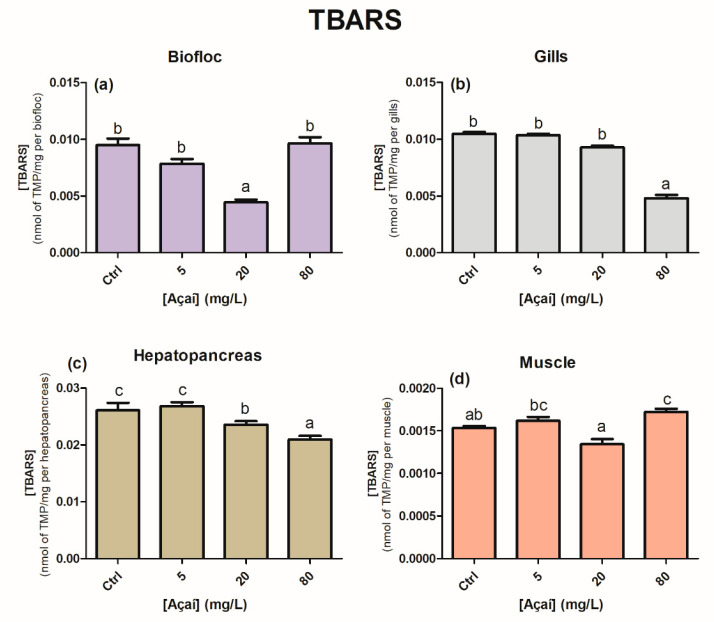
Levels of lipid peroxidation (nmol TMP/mg per tissue) in the bioflocs (**a**), gills (**b**), hepatopancreas (**c**), and muscle (**d**) of *Penaeus vannamei* shrimp after the addition of different concentrations of lyophilized açaí for 30 days. Values are expressed as mean ± 1 standard error (*n* = 12). Different letters indicate statistical differences between treatments according to the Newman–Keuls test at a significance level of 0.05. TMP is tetramethoxypropane, which was used as the standard.

The total antioxidant capacity (ACAP) of the bioflocs and hepatopancreas was lower (larger relative area) in treatments with 20 and 80 mg açaí L^−1^ and 5 and 20 mg açaí L^−1^, respectively, than in the control group (*p* < 0.05) (Figure 2a,c). No statistically significant differences were observed in the ACAP of the gills and muscles (*p* > 0.05) (Figure 2b,d). In relation to lipid peroxidation, the bioflocs, gills, and hepatopancreas showed a reduction in lipid damage (Figure 3). In bioflocs and gills, a decrease in lipid damage was observed at 20 and 80 mg of açaí L^−1^ compared to the other treatments (*p* < 0.05). In the hepatopancreas, a gradual decrease in lipid peroxidation was observed with increasing concentrations of lyophilized açaí (*p* < 0.05). There was an increase in lipid damage in the muscle in the 80 mg açaí L^−1^ treatment when compared to the control and 20 mg açaí L^−1^ treatments (*p* < 0.05).

**Figure 4:**
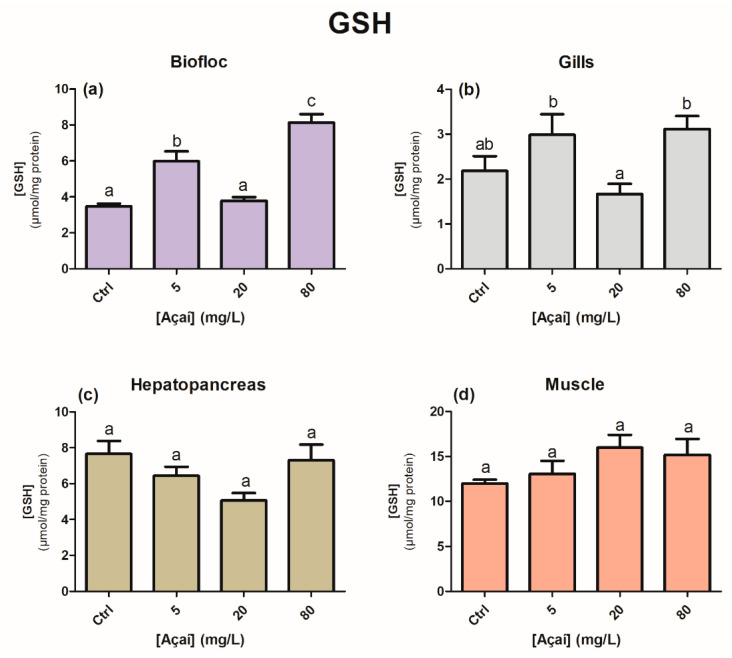
Concentration of reduced glutathione (GSH, μmol/mg protein) in the bioflocs (**a**), gills (**b**), hepatopancreas (**c**), and muscle (**d**) of *Penaeus vannamei* shrimp after the addition of different concentrations of lyophilized açaí for 30 days. Values are expressed as mean ± 1 standard error (*n* = 12). Different letters indicate statistical differences between treatments according to the Newman–Keuls test at a significance level of 0.05.

**Figure 5:**
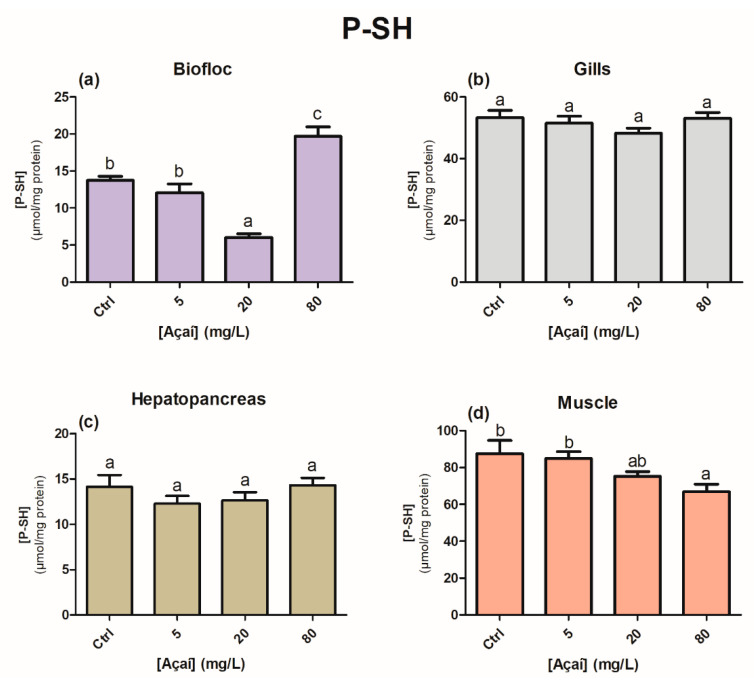
Concentration of protein sulfhydryl groups (μmol/mg protein) in the bioflocs (**a**), gills (**b**), hepatopancreas (**c**), and muscle (**d**) of *Penaeus vannamei* shrimp after the addition of different concentrations of lyophilized açaí for 30 days. Values are expressed as mean ± 1 standard error (*n* = 12). Different letters indicate statistical differences between treatments according to the Newman–Keuls test at a significance level of 0.05.

The GSH levels of the bioflocs increased significantly in the treatments with 5 and 80 mg açaí L^−1^, which differed from the control and 20 mg açaí L^−1^ groups (*p* < 0.05) (Figure 4a). In the gills, GSH levels differed only between treatments that received açaí (*p* < 0.05); however, when compared to the control treatment, there were no significant differences (Figure 4b). No significant differences were observed in the hepatopancreas or muscle (*p* > 0.05) (Figure 4c,d). The P-SH levels of the bioflocs increased at a concentration of 80 mg açaí L^−1^ (*p* < 0.05); however, in the muscle tissue, at the same concentration, there was a decrease in the mean values of P-SH compared to the control treatment (*p* < 0.05) (Figure 5a,d). No statistical differences were observed in the gill and hepatopancreas (*p* > 0.05) (Figure 5b,c).

**Figure 6:**
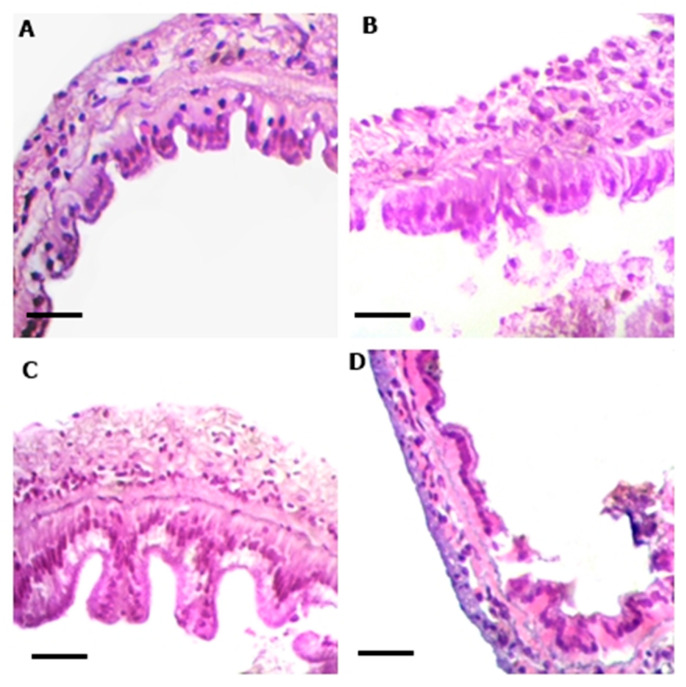
Middle intestine of juvenile shrimp *Penaeus vannamei* reared in a biofloc technology system (BFT) with different concentrations of açaí *Euterpe oleracea* for 30 days. (**A**)—Control; (**B**)—5 mg açaí L^−1^; (**C**)—20 mg açaí L^−1^; (**D**)—80 mg açaí L^−1^. H-E. BAR: 10 µm.

**Figure 7:**
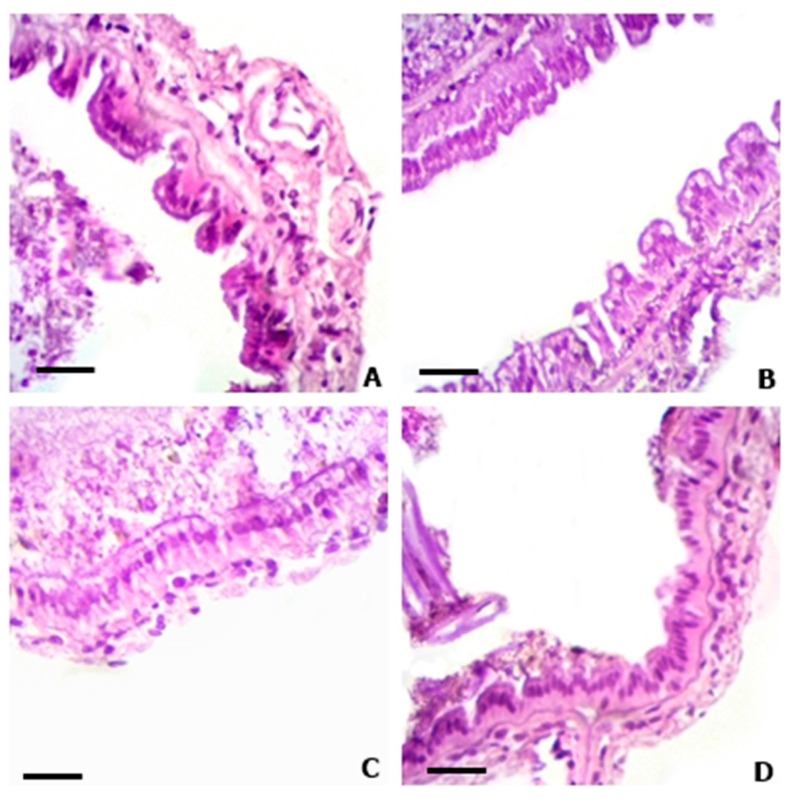
Posterior intestine of juvenile shrimp *Penaeus vannamei* reared in a biofloc technology system (BFT) with different concentrations of açaí *Euterpe oleracea* for 30 days. (**A**)—Control; (**B**)—5 mg açaí L^−1^; (**C**)—20 mg açaí L^−1^; (**D**)—80 mg açaí L^−1^. H-E. BAR: 10 µm.

The results of histological analysis are presented in Table 4 and Figure 6 and Figure 7. When evaluating the data from the middle intestine of shrimp, the addition of 5 and 20 mg açaí L^−1^ contributed to an increase in the mean height and area of the intestinal microvilli (*p* < 0.05). However, in the posterior intestine, the application of 20 and 80 mg açaí L^−1^ in the BFT system decreased the mean height and area of the microvilli compared to the control treatment (*p* < 0.05).

## 4. Discussion

The results of the water quality parameters of the present study highlighted that the daily addition of lyophilized açaí to the BFT system increased TSS levels; however, the mean values of total suspended solids remained below 500 mg/L, as recommended by Gaona et al. [31] (Table 1). The increase in açaí concentration in the 20 and 80 mg açaí L^−1^ treatments resulted in an increase in the pH and alkalinity of the water. Corrections with sodium bicarbonate (NaHCO_3_) to maintain alkalinity levels during the experiment were only necessary in the control and the 5 and 20 mg of açaí L^−1^ treatment groups, corroborating the recent study by Colombo et al. [27], where corrections were performed only in the control and 5 mg of açaí L^−1^ treatments during the cultivation of post-larvae of *P. vannamei* in a BFT system. Açaí, as well as other fruits and vegetables, has antioxidant and alkalizing potential, intensifying according to the lower degree of food processing [44,45]. Therefore, the inclusion of 80 mg açaí L^−1^ in the water for the cultivation of *P. vannamei* juveniles in the BFT system maintained the alkalinity levels during the experimental period.

The action of nitrifying bacteria in the BFT system is of fundamental importance for the success of their activity, because they efficiently cycle nitrogenous compounds generated during cultivation [19]. The results of the present study demonstrated that the daily addition of 80 mg açaí L^−1^ to *P. vannamei* cultivation in a BFT system increased ammonia levels and decreased nitrate concentrations (Table 1), corroborating the results observed by Colombo et al. [27]. Considering the protein content of açaí—8.1% to 10.5% [7,26]—ally to the ration provided, it is possible that the ingestion of both by shrimp in the 80 mg açaí L^−1^ treatment contributed to an increase in ammonia excretion [46,47,48]. Another hypothesis is the possible inhibition of the action of nitrifying bacteria owing to the action of antioxidants present in the fruit. Studies have shown that the application of plant extracts with antioxidant capacity in agricultural soils can produce a toxic effect on nitrifying bacteria of the genus *Nitrosomonas* by blocking the ammonia monooxygenase pathway, an enzyme that acts in the first stage of the conversion of ammonia to nitrite [49,50,51]. In aquaculture, this reflects higher levels of ammonia in the culture water, and consequently, lower concentrations of nitrite and nitrate. However, the results of the present study and those of Colombo et al. [27] only identified changes in the ammonia and nitrate concentrations. The presence of ammonia-oxidizing bacteria (AOB) and nitrite-oxidizing bacteria (NOB) nitrifying bacteria in the biofloc inoculum used in the experiment may have attenuated the inhibition of the ammonia monooxygenase pathway because ammonia was already converted to nitrate in the system. The addition of antioxidants during biofloc formation should be explored in future studies.

The ash levels in the bioflocs of the control treatments up to 20 mg açaí L^−1^ ranged from 45.61% to 40.47%, corroborating the results of Colombo et al. [27], who verified levels from 47.40% to 39.68% in the same treatments (Table 2). Similar values have been reported by Wasielesky et al. [21], Fernandes Da Silva et al. [52], and Reis et al. [53] that reported 44.85%, 45.50%, and 53.68% of ash in bioflocs from the cultivation of *P. vannamei* juveniles, respectively. According to studies by Silva et al. [26] and Odendaal and Schauss [7] the pulp of lyophilized açaí has an ash content of around 3.03% to 3.80%. In this context, the gradual decrease in the ash content of the bioflocs from the 20 mg açaí L^−1^ treatment was due to the accumulation of lyophilized açaí in the BFT system, which was incorporated into the biomass of the bioflocs.

Owing to their high protein content, bioflocs consumed by shrimp can replace a fraction of the required protein demand, thus complementing the artificial feed provided [21,54,55]. Considering the protein and lipid content of lyophilized açaí pulp [2,7], combined with the daily addition of this fruit to the artificial diet and the simultaneous production of bioflocs during the experimental period, we expected to verify an increase in the levels of protein and/or lipids in the bioflocs, as observed by Colombo et al. [27]; however, the differences were not statistically significant. The proximal composition of the muscle of juvenile *P. vannamei* also seemed to have been unaffected by the experimental treatments, although the protein content recorded was significantly higher in the 5 mg of açaí L^−1^ treatment compared to other treatments enriched with açaí, and did not differ from the control (Table 2).

In relation to bioflocs, the increase in açaí concentration influenced the loss of antioxidant capacity (larger relative area) (Figure 2a). Similar results were observed by Colombo et al. [27], where the enrichment of bioflocs with açaí for 27 days showed a dose–response behavior by decreasing the antioxidant capacity as the concentrations of this fruit increased. Quercetin, a phenolic compound with antioxidant properties, when applied to a culture of *P. vannamei* in a BFT system for 30 days also influenced the gradual loss of antioxidant capacity of the bioflocs [25]. In the present study, the loss of ACAP from the bioflocs may have influenced the decrease in the antioxidant capacity of the hepatopancreas (greater relative area) with the increase in açaí concentrations, mainly in the 5 and 20 mg açaí L^−1^ treatments (Figure 2a,c) because this organ is linked to the digestive process, secretion, and transport of several enzymes necessary for metabolism [54].

Although it lost its antioxidant capacity, the addition of 20 mg açaí L^−1^ prevented lipid peroxidation in the bioflocs, corroborating the results of Colombo et al. [27]; however, it was not sufficient to prevent oxidative damage to proteins in the same treatment group (Figure 3a). Peroxyl radicals are byproducts formed during lipid peroxidation; however, owing to their high antioxidant capacity, phenolic compounds can act in the interception of ROS, preventing the propagation of the lipoperoxidation process [56,57]. The protein damage observed in the bioflocs was related to the oxidation and decrease in the sulfhydryl groups (-SH) caused by the action of ROS (Figure 5a) [58].

The increase in height and area of the microvilli in the middle intestine of the shrimp was stimulated by the application of 20 mg açaí L^−1^, suggesting better intestinal absorption of the nutrients present in the açaí and compensating for their absorption in the posterior intestine (Table 4). The function of the posterior intestine is associated with the compaction of feces and the formation of the fecal cord rather than the absorption of nutrients [59,60]. Increasing the length and area of intestinal microvilli, in addition to expanding the nutrient absorption surface, can also improve osmoregulation and the immune system of shrimp [61,62,63]. The degree of intestinal absorption of flavonoids is related to their chemical structures. According to Lipinski et al. (2001) [64], compounds that are more easily absorbed by the intestine contain up to five H-bond donors and ten H-bond acceptors, molecular weights less than 500 Da, and an octanol:water partition coefficient lower than 5. However, molecules with many hydroxyls, glycosidic groups, and galloyl groups in their structure are difficult to absorb in the intestine [65]. The main flavonoids found in açaí are orientin, homoorientin, vitexin, luteolin, chrysoeriol, quercetin, and dihydrokaempferol [66]. Therefore, the intestinal absorption capacity of açaí depends on the chemical structure of the main flavonoids present in the fruit.

Regarding the zootechnical results, the mortality registered in the treatment group with 80 mg açaí L^−1^ can be attributed to the effects provoked by the high administration of açaí, which may have exerted a pro-oxidant condition for the cultivation of *P. vannamei* juveniles, and to the low absorption of nutrients in the middle intestine (Table 3 and Table 4). The impairment of zootechnial parameters has been reported by Xiong et al. (2022) [67], where high levels of the antioxidant lipoic acid in diets with low levels of carbohydrates (15%) resulted in lower weight gain and specific growth rate. Açaí pulp has a high content and diversity of molecules that, when metabolized, can act as pro-oxidants in different shrimp organs [10]. The growth phases of *P. vannamei* cultivated in a BFT system enriched with açaí also seem to influence its oxidant state in different ways, as the survival of the post-larvae of the same species was affected by the addition of 80 mg açaí L^−1^ [27].

When analyzing biochemical data from the muscle of shrimp cultivated in the 80 mg açaí L^−1^ treatment, no differences were observed in ACAP and GSH levels (Figure 2d and Figure 4d). In the muscles of juvenile *P. vannamei* exposed to nano-encapsulated lipoic acid in a BFT system, there were no statistical differences in GSH levels [24]. The lack of response regarding antioxidant defenses may have aggravated the occurrence of protein and lipid oxidative damage in the muscle, contributing to the low survival (Figure 3d and Figure 5d). Despite the high mortality that occurred in the 80 mg açaí L^−1^ treatment, the antioxidant effects of lyophilized açaí in terms of a reduction in lipid peroxidation were evident in the gills and hepatopancreas (Figure 3b,c).

## 5. Conclusions

The 20 and 80 mg açaí L^−1^ concentrations acted as natural alkalinizers in the BFT system by maintaining water alkalinity levels above 100 mg CaCO_3_ L^−1^. In relation to nitrogenous compounds, more studies are needed to clearly demonstrate whether high concentrations of açaí in a biofloc system can interfere with nitrification. The application of up to 20 mg açaí L^−1^ was able to minimize oxidative damage in the bioflocs and organs of the shrimp, as well as to stimulate an increase in the height and area of the intestinal microvilli. The results demonstrated that the high mortality in the treatment group with 80 mg açaí L^−1^ in the BFT system was due to the pro-oxidant action of açaí and the low intestinal absorption capacity of the shrimp. It is concluded that the bioflocs were able to assimilate the antioxidants present in the açaí and transfer them to the shrimp, and among the different concentrations of açaí, the administration of 20 mg açaí L^−1^ presented the best performance in terms of biochemical parameters and histological studies, demonstrating the possibility of its application in the cultivation of *P. vannamei* in a biofloc system.

## Figures and Tables

**Table 1:** Physicochemical parameters of water during shrimp *Penaeus vannamei* reared in the biofloc technology system (BFT) with different concentrations of açaí *Euterpe oleracea***.** Values are expressed as mean ± 1 standard error (*n* = 3).

Parameters	Treatments			
	Control	5 mg L^−1^	20 mg L^−1^	80 mg L^−1^
Dissolved oxygen (mg L^−1^)	6.13 ± 0.03 ^a^	6.25 ± 0.04 ^a^	6.19 ± 0.04 ^a^	6.16 ± 0.03 ^a^
Temperature	29.85 ± 0.06 ^a^	29.67 ± 0.07 ^a^	29.84 ± 0.07 ^a^	29.84 ± 0.05 ^a^
pH	7.93 ± 0.009 ^a^	7.92 ± 0.008 ^a^	7.97 ± 0.008 ^b^	8.01 ± 0.005 ^c^
Ammonia (mg TAN L^−1^)	0.31 ± 0.05 ^a^	0.30 ± 0.04 ^a^	0.32 ± 0.04 ^a^	0.71 ± 0.06 ^b^
Nitrite—N (mg L^−1^)	0.16 ± 0.02 ^a^	0.16 ± 0.02 ^a^	0.21 ± 0.04 ^a^	0.27 ± 0.05 ^a^
Nitrate—N (mg L^−1^)	44.95 ± 4.61 ^a^	40.78 ± 3.68 ^a^	32.51 ± 2.72 ^a^	17.63 ± 1.48 ^b^
Phosphorus—P (mg L^−1^)	2.06 ± 0.35 ^a^	2.16 ± 0.37 ^a^	1.86 ± 0.28 ^a^	1.70 ± 0.28 ^a^
Alkalinity (mg CaCO_3_ L^−1^)	113.13 ± 5.09 ^a^	135.63 ± 4.77 ^a^	159.69 ± 6.02 ^b^	197.50 ± 5.31 ^c^
Salinity	26.48 ± 0.31 ^a^	26.65 ± 0.27 ^a^	27.05 ± 0.36 ^a^	26.55 ± 0.34 ^a^
TSS (mg L^−1^)	213.89 ± 16.98 ^a^	266.85 ± 24.88 ^b^	323.52 ± 30.05 ^c^	341.11 ± 25.38 ^c^

Different letters on the same line indicate significant differences between treatments according to the Newman–Keuls test at a significance level of 0.05.

**Table 2:** Proximal composition of the biofloc and muscle of *Penaeus vannamei* shrimp reared in the biofloc technology system (BFT) with different concentrations of açaí *Euterpe oleracea*. Values are expressed as mean ± 1 standard error (*n* = 6).

Parameters	Treatments			
	Control	5 mg L^−1^	20 mg L^−1^	80 mg L^−1^
**(A) Biofloc**				
Dry matter (%)	14.64 ± 0.82 ^a^	13.16 ± 0.86 ^a^	12.09 ± 0.19 ^a^	13.91 ± 0.21 ^a^
Protein (%)	30.02 ± 0.57 ^a^	29.56 ± 0.42 ^a^	27.73 ± 0.36 ^a^	32.27 ± 1.58 ^a^
Ether extract (%)	2.62 ± 0.65 ^a^	2.70 ± 0.49 ^a^	4.01 ± 0.95 ^a^	4.27 ± 0.33 ^a^
Ash (%)	45.61 ± 0.26 ^c^	44.92 ± 0.30 ^c^	40.47 ± 0.69 ^b^	27.74 ± 0.88 ^a^
**(B) Muscle**				
Dry matter (%)	23.59 ± 0.27 ^a^	23.47 ± 0.24 ^a^	24.04 ± 0.33 ^a^	25.13 ± 0.59 ^a^
Protein (%)	76.23 ± 1.12 ^ab^	77.75 ± 0.44 ^b^	74.44 ± 1.35 ^a^	74.80 ± 0.92 ^a^
Ether extract (%)	2.58 ± 0.34 ^a^	1.95 ± 0.39 ^a^	3.68 ± 0.85 ^a^	3.33 ± 0.21 ^a^
Ash (%)	6.26 ± 0.09 ^a^	6.42 ± 0.07 ^a^	6.37 ± 0.08 ^a^	5.59 ± 0.34 ^a^

Different letters on the same line indicate significant differences between treatments according to the Newman–Keuls test at a significance level of 0.05.

**Table 3:** Zootechnical parameters of *Penaeus vannamei* shrimp reared in the biofloc technology system (BFT) with different concentrations of açaí *Euterpe oleracea*. Values are expressed as mean ± 1 standard error (*n* = 31). Abbreviations: SGR—specific growth ratio; FCR—feed conversion ratio; PER—protein efficiency ratio.

Parameters	Treatments			
	Control	5 mg L^−1^	20 mg L^−1^	80 mg L^−1^
Initial weight (g)	1.09 ± 0.04 ^a^	1.10 ± 0.04 ^a^	1.06 ± 0.05 ^a^	0.96 ± 0.04 ^a^
Final weight (g)	3.95 ± 0.16 ^a^	4.14 ± 0.17 ^a^	3.80 ± 0.16 ^a^	3.56 ± 0.30 ^a^
Weight gain (g)	2.83 ± 0.15 ^a^	2.97 ± 0.17 ^a^	2.78 ± 0.16 ^a^	2.65 ± 0.30 ^a^
SGR (%/day)	4.05 ± 0.13 ^a^	4.06 ± 0.15 ^a^	4.03 ± 0.13 ^a^	3.98 ± 0.32 ^a^
FCR	2.00 ± 0.10 ^a^	2.27 ± 0.15 ^a^	2.27 ± 0.13 ^a^	1.90 ± 0.26 ^a^
PER	1.52 ± 0.08 ^a^	1.47 ± 0.08 ^a^	1.47 ± 0.09 ^a^	1.91 ± 0.21 ^a^
Survival (%)	79.57 ± 18.84 ^b^	92.47 ± 7.53 ^b^	88.45 ± 3.61 ^b^	38.71 ± 8.53 ^a^

Different letters on the same line indicate significant differences between treatments according to the Newman–Keuls test at a significance level of 0.05.

**Table 4:** Histological analysis of the middle and posterior intestines of shrimp *Penaeus vannamei* reared in a biofloc technology system (BFT) with different concentrations of açaí *Euterpe oleracea*. Values are expressed as mean ± 1 standard error (*n* = 12). Different letters on the same line indicate significant differences between treatments according to the Newman–Keuls test at a significance level of 0.05.

Treatments	Middle Intestine			Posterior Intestine		
	Width (µm)	Height (µm)	Area (µm^2^)	Width (µm)	Height (µm)	Area (µm^2^)
Control	32.70 ± 2.15 ^a^	24.10 ± 2.91 ^a^	674.34 ± 101.13 ^a^	34.86 ± 3.51 ^a^	29.49 ± 1.73 ^a^	903.32 ± 127.90 ^c^
5 mg L^−1^	36.50 ± 3.45 ^a^	36.99 ± 4.31 ^b^	1311.42 ± 223.48 ^b^	35.92 ± 3.91 ^a^	27.26 ± 2.88 ^a^	819.45 ± 111.33 ^bc^
20 mg L^−1^	40.89 ± 3.05 ^a^	50.64 ± 3.70 ^c^	1981.28 ± 212.50 ^c^	26.77 ± 2.76 ^a^	19.59 ± 1.84 ^b^	532.58 ± 98.49 ^ab^
80 mg L^−1^	31.64 ± 2.06 ^a^	28.31 ± 2.70 ^ab^	921.03 ± 149.19 ^ab^	28.66 ± 1.65 ^a^	16.18 ± 0.58 ^b^	443.39 ± 33.26 ^a^

Different letters on the same line indicate significant differences between treatments according to the Newman–Keuls test at a significance level of 0.05.

## Data Availability

The data presented in this study are openly available in FigShare at https://doi.org/10.6084/m9.figshare.23671809.v1 (accessed 12 October 2023).
